# Quantifying the contribution of bent shoots to plant photosynthesis and biomass production of flower shoots in rose (*Rosa hybrida*) using a functional–structural plant model

**DOI:** 10.1093/aob/mcz150

**Published:** 2019-09-24

**Authors:** Ningyi Zhang, Arian van Westreenen, Jochem B Evers, Niels P R Anten, Leo F M Marcelis

**Affiliations:** 1 Horticulture and Product Physiology Group, Department of Plant Sciences, Wageningen University, 6700 AA Wageningen, The Netherlands; 2 Centre for Crop Systems Analysis, Department of Plant Sciences, Wageningen University, 6700 AK Wageningen, The Netherlands

**Keywords:** Bent shoots, biomass allocation, functional–structural plant model, heterogeneous canopy, light absorption, photosynthesis, rose (*Rosa hybrida*)

## Abstract

**Background and Aims:**

The success of using bent shoots in cut-rose (*Rosa hybrida*) production to improve flower shoot quality has been attributed to bent shoots capturing more light and thus providing more assimilates for flower shoot growth. We aimed at quantifying this contribution of photosynthesis by bent shoots to flower shoot growth.

**Methods:**

Rose plants were grown with four upright flower shoots and with no, one or three bent shoots per plant. Plant architectural traits, leaf photosynthetic parameters and organ dry weight were measured. A functional–structural plant (FSP) model of rose was used to calculate photosynthesis of upright shoots and bent shoots separately, taking into account the heterogeneous canopy structure of these plants.

**Key Results:**

Bent shoots contributed to 43–53 % of total assimilated CO_2_ by the plant. Plant photosynthesis increased by 73 and 117 % in plants with, respectively, one and three bent shoots compared with plants without bent shoots. Upright shoot photosynthesis was not significantly affected by the presence of bent shoots. However, upright shoot dry weight increased by 35 and 59 % in plants with, respectively, one and three bent shoots compared with plants without bent shoots. The increased upright shoot dry weight was entirely due to the contribution of extra photosynthesis by bent shoots, as this was the only source that could induce differences in upright shoot growth apart from their own photosynthesis. At least 47–51 % of the photosynthesis by bent shoots was translocated to upright shoots to support their biomass increase.

**Conclusions:**

Based on model simulations, we conclude that the positive effect of shoot bending on flower shoot growth and quality in cut-rose production system can almost entirely be attributed to assimilate supply from bent shoots. FSP modelling is a useful tool to quantify the contributions of photosynthesis by different parts of heterogeneous canopies.

## INTRODUCTION

Rose (*Rosa hybrida*) is one of the most popular ornamental crops worldwide. In cut-rose production, weak and non-flowering shoots are usually bent downwards (so-called bent shoots; Supplementary data Fig. S1) to intercept light not captured by the upright shoots, which are the economically valuable flower shoots (Ohkawa and Suematsu 1997). Bending part of the shoots in rose plants increases stem length, flower size and dry weight of upright shoots, resulting in high commercial quality of harvestable flower shoots (Kool and Lenssen, 1997; Warner and Erwin, 2002; Särkkä and Eriksson, 2003; Kim and Lieth, 2004). The advantages of bent shoots in cut-rose production have been attributed to the extra assimilates produced by bent shoots from which growth of the upright flower shoots benefits (Kim and Lieth, 2004). However, bent shoots are lower down in the canopy and only receive a limited amount of light. Keeping too many bent shoots may therefore lead to a negative carbon balance especially in the lower layers of bent shoots (Pien *et al*., 2001), resulting in competition between bent shoots and upright shoots for assimilates produced by the whole plant canopy. To optimize the number of bent shoots and to maintain upright shoot quality, it is imperative to quantify to what extent photosynthesis by bent shoots may contribute to upright shoot photosynthesis and biomass production, and how this depends on bent shoot number. No studies have ever quantified such contributions.

Bent shoots can directly and indirectly contribute to upright shoot growth. The assimilates produced by bent shoots can directly contribute to upright shoot growth when they are translocated to the upright shoots (Baille *et al*., 2006; Kajihara *et al*., 2009). Photosynthesis of upright shoots themselves can also be indirectly affected by bent shoots. Upright shoot photosynthesis is determined by both upright shoot light interception and leaf photosynthetic characteristics. Although leaf photosynthetic characteristics of upright shoots are unlikely to be affected by bent shoots (Kim *et al*., 2004; González-Real *et al*., 2007), upright shoot light interception may be affected by the presence of bent shoots. The assimilate supply from bent shoots may enhance the establishment of leaf area in upright shoots and result in an increased light interception and photosynthesis.

To quantify the direct and indirect contributions of bent shoots to upright shoot growth, a crucial step is to separately quantify both contributions to plant photosynthesis in the canopy, as photosynthesis provides assimilates for plant growth. This contribution is difficult to derive experimentally but can be done using simulation models. However, most canopy photosynthesis models (e.g. multilayer, big-leaf or sun-shade models) assume an even light distribution in the canopy (Hikosaka *et al*., 2016b). Since the rose plants consist of vertically growing upright shoots and horizontally growing bent shoots, they constitute a spatially heterogeneous canopy, which cannot be represented in conventional canopy photosynthesis models to calculate canopy photosynthesis. This issue can be solved by using functional–structural plant (FSP) models. In FSP models, individual plants and their architecture and functioning in a canopy are represented in three dimensions (Vos *et al*., 2010), which has been applied to rose before (Buck-Sorlin *et al*., 2011). This approach can therefore be applied to calculate photosynthesis of a heterogeneous rose crop at the leaf level.

The objective of this study was to quantify the relative contributions of bent shoots and upright shoots to photosynthesis of the whole plant. To this end, first a greenhouse experiment with rose plants was conducted to investigate the effects of bent shoots on upright shoot architectural development, leaf photosynthesis and biomass production. The plants were subjected to bending treatments whereby different numbers of bent shoots (no, one or three shoots) were retained on the plant and four shoots were retained to grow vertically (the so-called upright shoots). Upright shoot morphology, leaf photosynthesis and organ biomass were measured. Then, an FSP model of rose was developed based on morphology and photosynthesis measurements, and was used to calculate photosynthesis of bent shoots and upright shoots for plants in the different bending treatments.

## MATERIALS AND METHODS

A combination of experimentation and model simulation was applied in this study. First, an experiment was conducted in which each plant had either no, one or three bent shoots. This experiment was used to study the effects of bent shoots on flower shoot architecture and biomass production. From this experiment, plant architectural and leaf photosynthetic data were collected to develop and validate a 3-D rose model. Then, the rose model was used to calculate plant daily photosynthesis by bent shoots and upright shoots during the experimental period, based on the actual hourly light intensity in the greenhouse.

### Experimentation

#### Plant growth conditions

Rose plants (*Rosa hybrida* ‘Red Naomi!’) were grown in a compartment (12 × 12 × 4 m) of a Venlo-type glasshouse located in Wageningen, The Netherlands (52°N, 6°E). The compartment contained six growth beds each consisting of two gutters. On these gutters, rooted rose cuttings bearing a shoot (the primary shoot) were planted on 4 January 2017 at a plant density of 7.5 plants m^–2^ to closely resemble a realistic commercial cultivation set-up (Supplementary data Fig. S1A). On 24 January 2017 when all primary shoots had formed a flower bud, the flower buds were removed (Supplementary data Fig. S1B). On 6 February 2017 when all primary shoots had developed side shoots on the top, these primary shoots were bent downwards (primary bent shoots, hereafter) (Supplementary data Fig. S1C). This mimics the primary bending done in practice. After bending the primary shoots, on average two axillary buds per plant sprouted. These newly sprouted shoots were harvested on 9 March 2017 because they were too thick to bend. After the harvest, on average four axillary buds per plant sprouted, with two axillary buds on each parent stem. When these axillary buds had developed into mature shoots, two of these shoots were bent downwards (secondary bent shoots, hereafter) on 5 April 2017. The other two shoots were harvested on 12 April 2017 (Supplementary data Fig. S1D). After this harvest, four axillary buds on each plant were allowed to grow upwards (upright shoots, hereafter) to become the economically valuable cut-flower products (Supplementary data Fig. S1E).

High-pressure sodium (HPS) lamps (600W, Philips, Eindhoven, The Netherlands) were used between 2.00 and 21.00 h, only when global radiation outside the greenhouse dropped below 200 W m^–2^ and were switched off when outside global radiation increased to values >300 W m^–2^. Light intensity from the HPS lamps when they were on was approx. 150 μmol m^–2^ s^–1^ at canopy level. The shading screen (HARMONY 4215 O FR, Ludvig Svensson, Hellevoetsluis, The Netherlands) was closed when outside global radiation increased to values >600 W m^–2^ and was opened when outside global radiation dropped below 500 W m^–2^. CO_2_ was supplied when the CO_2_ concentration inside the greenhouse was <700 ppm, which is similar to commercial greenhouse settings. However, when windows were opened, CO_2_ supply stopped. The average light intensity [photosynthetically active radiation (PAR) from both sun and lamps] during the photoperiod (02.00–21.00 h) inside the greenhouse throughout the experiment (from 24 April to 31 May 2017) was 360 μmol m^–2^ s^–1^. The average daily temperature, relative humidity and CO_2_ concentration inside the greenhouse during the experiment were 21.6 °C, 72 % and 524 ppm, respectively. Plants were irrigated hourly between 07.00 and 19.00 h with standard nutrient solution (EC = 2.2 mS cm^–1^; pH = 5.8) for rose crop used in practice.

#### Treatments

On 24 April 2017, treatments started. At that point, each plant had four axillary buds and three bent shoots (including one primary bent shoot and two secondary bent shoots). In total three treatments were established: (1) all bent shoots were removed from the plants (0B); (2) the secondary bent shoots were removed from the plants and the primary bent shoots were kept on the plants, resulting in one bent shoot per plant (1B); and (3) all bent shoots were kept on the plants, resulting in three bent shoots per plant (3B). In all treatments, four axillary buds were kept, resulting in four upright flower shoots per plant. On day 38 after the start of treatment, all upright shoots were harvested when they were blooming, and the experiment ended.

#### Plant architecture and biomass measurements

Six plants per plot (in total 18 plants per treatment) were randomly chosen and one upright shoot on each plant was used to measure architectural traits and biomass production. Shoot architectural traits were measured non-destructively on days 6, 9, 14, 19 and 25 after the start of treatment. The non-destructive measurements included the number of leaves on the shoot, length and width of all individual leaves on the shoot, stem length, length of all internodes on the shoot, and flower width. On day 37 after the start of treatment, all upright shoots used for non-destructive measurements were harvested for destructive measurements. The destructive measurements included shoot fresh weight, stem length, length and diameter of all internodes on the shoot, length, width, leaflet number, area and inclination angle of all individual leaves on the shoot, and flower width. An allometric relationship between leaf area and the product of leaf length and width was derived from the destructive measurements (*r*^2^ = 0.92; relative root-mean-square error = 0.13), and was used to calculate leaf areas on days when non-destructive measurements on leaf length and width were performed. The length of the compound rose leaf was measured from the tip of the terminal leaflet to the end of the petiole where the leaf was attached to the stem, and leaf width was measured at the widest part of the leaf. After the destructive measurements, individual organs were put in an oven for 48 h at 105 °C to measure organ dry weight. Stem length was measured using a measuring tape. Leaf length and width, internode length and flower width were measured using a ruler. Internode diameter was measured using a pair of callipers. Leaf area was measured using a leaf area meter (LICOR-3100; Li-Cor BioScience, Lincoln, NE, USA). Leaf inclination angle was measured as the insertion angle of the leaf with the horizontal level using a protractor. Shoot fresh weight was measured using a common electronic balance. Organ dry weight was measured using an analytic balance.

In addition, for the 18 plants used for upright shoot measurements in each treatment, total leaf area and shoot dry weight of bent shoots were measured. As the 0B and 1B treatments entailed removal of bent shoots at the start of treatment, one of the removed bent shoots per plant was sampled for measurements. At the end of the experiment, one bent shoot per plant was sampled in the 1B and 3B treatments.

#### Light measurements

On days 13 and 30 after the start of treatment (both were overcast days), light measurements were performed using a line quantum sensor (Li-Cor BioScience). In each plot, measurements were performed at three locations (at the front, middle and back of the plot; the distance between the front and middle and between the middle and back was 60 cm). At each location, light intensities were measured above, in the middle of and at the bottom of upright shoots, and above and below bent shoots.

#### Leaf gas exchange measurements

At the flowering stage, two plants per plot were chosen from the six plants used for architecture and biomass measurements. One shoot per plant was chosen to perform a combined measurement of leaf gas exchange and chlorophyll fluorescence using the LI-6400XT Portable Photosynthesis System (Li-Cor BioScience). The measurements were performed on terminal leaflets of leaves at the upper, middle and lower level of the upright shoots and leaves on the bent shoots. Light response curves of photosynthesis were made by decreasing incident light in the leaf cuvette in the series of 1500, 1100, 700, 400, 200, 150, 100, 75 and 50 μmol m^–2^ s^–1^, while keeping ambient CO_2_ at 400 μmol mol^–1^, O_2_ at 21 %, leaf temperature at 25 °C and leaf to air vapour pressure difference at 1–1.6 kPa. The steady-state fluorescence (*F*_s_) was measured simultaneously with the gas exchange measurement after 3–5 min light adaptation. The maximum fluorescence *F*_m_’ was measured using the multiphase flash method. The flash intensity (*I*_flash_) was increased from the background light level in the leaf cuvette to approx. 6300 μmol m^–2^ s^–1^ in phase 1, and *I*_flash_ was maintained at approx. 6300 μmol m^–2^ s^–1^ for 300 ms; then *I*_flash_ was decreased by 35 % and maintained at this level for 300 ms in phase 2; in phase 3, *I*_flash_ was back to the level of phase 1 and was maintained at this level for 300 ms. The intercept of linear regression of fluorescence yields during phase 2 against 1/*I*_flash_ gives the estimate of *F*_m_’ from the multiphase flash method (Loriaux *et al*., 2013).

#### Estimating leaf photosynthetic parameters

To study the effect of shoot bending on leaf photosynthetic traits of upright shoots and bent shoots, leaf photosynthetic parameters were estimated based on measured light response curves. Three photosynthetic parameters [maximum leaf photosynthetic rate *A*_max_, dark respiration rate *R*_d_ and quantum yield *Φ*_CO2LL(inc)_] were estimated for leaves of upright shoots and bent shoots in each treatment. Photosynthetic parameters were estimated by stepwise fitting the combined measurements of gas exchange and chlorophyll fluorescence to a non-rectangular hyperbola [eqn (1)] (Marshall and Biscoe, 1980):

$$\begin{array}{l} A=\frac{\Phi_{CO2LL(inc)}I_{inc}+A_{max}-\sqrt{{\left(\Phi_{CO2LL(inc)}I_{inc}+A_{max}\right)}^{2}-4\theta A_{max}\Phi_{CO2LL(inc)}I_{inc}}}{2\theta }-R_{d} \end{array}$$ (1)

where *A* (μmol CO_2_ m^-2^ s^-1^) is the net leaf photosynthetic rate; *Φ*_CO2LL(inc)_ (mol CO_2_ mol^–1^ photon) is the quantum yield of CO_2_ assimilation on the basis of incident light; *I*_inc_ (μmol m^–2^ s^–1^) is the incident light; *A*_max_ (μmol CO_2_ m^–2^ s^–1^) is the maximum leaf photosynthetic rate at saturating incident light; *θ* is the curvature factor of the light response curve and was kept at 0.8 (Yin and Struik, 2015); and *R*_d_ (μmol CO_2_ m^–2^ s^–1^) is the dark respiration rate. Details on the procedure of estimating *Φ*_CO2LL(inc)_, *A*_max_ and *R*_d_ can be found in Supplementary data Method S1.

To further calculate daily photosynthesis using the rose model, photosynthetic parameters of each individual leaf on the plant need to be determined. Generally in a canopy, *A*_max_ distribution of individual leaves can be assumed to follow light distribution along the canopy. This assumption is largely based on the premise that light distribution in the canopy drives leaf nitrogen distribution, which in turn determines the distribution of *A*_max_ (Hirose and Werger, 1987; Hikosaka, 2014). As the extinction coefficients of leaf nitrogen in upright shoots and in bent shoots are similar (González-Real *et al*., 2007), we assumed the same correlation between the light gradient and the *A*_max_ gradient in both upright and bent shoots. To derive photosynthetic parameters of individual leaves in both upright and bent shoots, first the relationship between the light gradient and the *A*_max_ gradient in upright shoots was quantified using eqn (2) (Niinemets and Anten, 2009; Hikosaka *et al*., 2016b):

$$\begin{array}{l} A_{max}=A_{0}\times {\left(Q/Q_{0}\right)}^{k} \end{array}$$ (2)

where *A*_max_ (μmol CO_2_ m^–2^ s^–1^) is the estimated *A*_max_ for a leaf; *A*_0_ (μmol CO_2_ m^–2^ s^–1^) is the photosynthetic capacity of the highest most illuminated leaf in upright shoots (*A*_0,upright_); *Q* (μmol m^–2^ s^–1^) is the measured light intensity at the level of photosynthesis measurements for estimating *A*_max_; *Q*_0_ (μmol m^–2^ s^–1^) is the measured light intensity above the plants; and *k* is the coefficient determining the relationship between the light gradient and the *A*_max_ gradient in a canopy. Taking the logarithm of both sides of eqn (2) gives a linear equation [eqn (3)]:

$$\begin{array}{l} log\;A_{max}=log\;A_{0}+k\times log\;\left(Q/Q_{0}\right) \end{array}$$ (3)

By curve fitting eqn (3) with values of *A*_max_ and *Q*/*Q*_0_ obtained from measurements on the top, middle and lower leaves of upright shoots, *A*_0,upright_ and *k* were derived. For bent shoots, the relationship between the light gradient and the *A*_max_ gradient was also quantified using eqn (3). In this case, *A*_0_ [in eqns (2) and (3)] is the photosynthetic capacity of the highest most illuminated leaf in bent shoots (*A*_0,bent_). The *k* value is kept at the same value estimated for upright shoots. *A*_0,bent_ was then derived by fitting the intercept of eqn (3) with values of *A*_max_ and *Q*/*Q*_0_ obtained from leaves of bent shoots. According to the values of *A*_0,upright_, A_0,bent_ and *k*, and the relative light level experienced by a leaf, *A*_max_ of that leaf can be derived using eqn (2).

#### Statistical analysis

The treatments were established with a randomized block design, with three blocks and 72 plants per plot. In the analysis, the treatments were considered as independent fixed factors. The treatment effects on upright shoot architectural traits, upright shoot fresh weight, upright shoot and organ dry weight, upright shoot biomass allocation and leaf photosynthetic parameters were analysed using a one-way analysis if variance (ANOVA; *P* < 0.05) of R (version R 3.3.3, R Core Team).

### Simulation

#### Model development

A 3-D rose model was constructed in the plant modelling software GroIMP (Hemmerling *et al*., 2008). The model includes (1) a 3-D representation of rose plant architecture that changes every 3 d; (2) a radiation and photosynthesis model to calculate plant light absorption and photosynthesis; and (3) virtual sensors to measure light intensities in the canopy analogous to the measurements done in the experiment.

##### (1) 3-D rose plants.

Three-dimensional representations of rose plants were constructed for the three treatments. Each plant was composed of four upright shoots, and no, one or three bent shoots. Upright shoots were constructed using phytomers consisting of an internode and a compound rose leaf, that together make up the shoot. Architectural parameters used for constructing each individual upright shoot included length and width of all internodes, length, width, leaflet number and inclination angle of all leaves, and flower width. Architectural measurements (measured every 3–5 d) were used to build a database which contains sets of individual architectural parameters. Each parameter set was obtained from architectural measurements of one shoot. In simulations, the architecture of individual shoots was changed every 3 d (from day 1 to dasy 25 after the start of treatment) according to the parameter sets that were randomly selected from the database. This time step was chosen to match the measurement frequency, which was selected based on the shoot growth rate in the greenhouse. In cases where the architectural measurements were not performed on that day, parameters were derived from linear interpolations of parameter values measured on the two closest days. From day 25, the architectural parameters no longer changed, as parameter values measured on day 25 were similar to the final measurements on day 35. Due to the architectural complexity of bent shoots (Supplementary data Fig. S2), it is nearly impossible to document the distributions of leaf area and leaf angle for bent shoots. Therefore, we adopted the spherical leaf angle distribution which assumes that all orientations of the leaf surfaces have equal probability (i.e. a random leaf angle distribution) (Goudriaan, 1988), and constructed the bent shoots by randomly distributing a number of leaves in the area occupied by bent shoots. Sensitivity analysis showed that the random leaf angle distribution gave very similar light interception results compared with a fully horizontal distribution of the leaves (Supplementary data Figs S3 and S4). In addition, as the bent shoot leaf area was relatively high in our experiment, the distribution of leaf angles had a relatively small effect on light interception. The difference of light interception between a totally vertical and horizontal leaf angle distribution (the two most contrasting situations) was <10 % (Supplementary data Fig. S4). Total leaf areas of bent shoots used for treatments with one (1B) and three (3B) bent shoots were obtained from the experiment. For the treatment with no bent shoots (0B), simulations were performed without bent shoots. Plant density was set to the same values as in the experiment to mimic the actual plant arrangements in the experiment.

##### (2) The radiation and photosynthesis model.

The light environment was modelled using a dome of light sources representing diffuse light emitted by an overcast sky with moderate gradation towards zenith and azimuthal uniformity (Evers *et al*., 2010). To eliminate the border effects in the light environment, the simulated plant population (in total 18 plants were simulated, with two rows and nine plants in each row) was replicated ten times in the *x* and *y* directions, resulting in average light conditions as experienced by 100 copies of each individual plant population (1800 plants in total) (de Vries *et al*., 2018). The amount of light reaching the plant organs was simulated using a Monte Carlo ray tracer (with 5 million rays) embedded in GroIMP (Hemmerling *et al*., 2008). The light reflectance (= 0.08) and transmittance (= 0.06) values of rose leaves were obtained from spectrophotometric measurements on rose leaves of the same cultivar in another experiment; stems were assumed to have the same reflectance as leaves but with no transmission. Plant net photosynthesis was calculated as the sum of net photosynthesis of individual leaves, which was in turn calculated based on the light absorption and photosynthetic parameters of individual leaves [see eqn (1)]. *A*_max_ of individual leaves in upright shoots and bent shoots was derived based on the relative light intensity [*Q*/*Q*_0_ in eqn (2)] experienced by that leaf and parameter values of *A*_0,upright_, *A*_0,bent_ and *k*. *R*_d_ of individual leaves was assumed to be proportional to *A*_max_ of that leaf (Hikosaka *et al*., 2016b). All leaves were assumed to have the same quantum efficiency [*Φ*_CO2LL(inc)_] as we found that neither the treatments nor the positions of the leaves in the canopy affected this parameter. The stem surface area was not taken into account for the photosynthesis calculation despite its potential contribution to shoot photosynthesis, because (*a*) the proportion of the stem surface area to leaf area of a shoot was rather small (approx. 2%); (*b*) the projection area of the stem surface was low in upright shoots due to the fact that the orientation of the stem surface was largely parallel to the direction of incoming light, whereas stems of bent shoots were largely shaded by leaves; and (*c*) it was hard to determine the photosynthetic parameters that should be used for the stem, as we did not have reliable measurements for the stem photosynthesis.

##### (3) Virtual sensors. 

Virtual light sensors were constructed such that the light intensity could be monitored similar to the line quantum sensor used in the experiment. Virtual sensors were placed at the same locations as where actual light measurements were performed in the experiment.

#### Calculating light intensity inside the greenhouse for model input.

Global radiation was documented every 5 min by a weather station located outside the greenhouse. The use of the shading screen and artificial lighting inside the greenhouse was also documented every 5 min. Based on the global radiation, the overall transmission of the greenhouse (= 0.6), transmission of the shading screen (= 0.7), the artificial light intensity at the canopy level (= 150 μmol m^–2^ s^–1^) and the durations of applying the shading screen and the artificial lighting, light intensity inside the greenhouse was calculated every 5 min. These values were used to calculate the average light intensity inside the greenhouse (including light from both sun and lamps) during the experiment for model evaluation, and to calculate the hourly light intensity inside the greenhouse during the light period for simulation studies.

#### Model evaluation

Light measurements by virtual sensors were performed for the three treatments using 3-D representations of plant architecture on days 14 and 25 after the start of treatments. The incoming light intensity in the model was kept at 360 μmol m^–2^ s^–1^, which represented the average light intensity inside the greenhouse during the experiment. Light measurements by virtual sensors were compared with the actual light measurements on days 13 and 30 after the start of treatment. Since the incoming light intensity varied during the actual measurements, light intensities relative to that above upright shoots were used for model evaluation. Relative light intensities obtained in the experiment were compared with simulations by calculating the coefficient of determination (*r*^2^) and the relative root-mean-square error (rRMSE):

$$\begin{array}{l} rRMSE=\;\;\;\frac{1}{\bar{x}}\sqrt{\frac{\overset{n}{\underset{i=1}{\sum }}{\left(y_{i}-x_{i}\right)}^{2}}{n}} \end{array}$$ (4)

where *y*_*i*_ is the simulated value, *x*_*i*_ is the measured value, *n* is the number of data points and $\bar{x}$ is the mean of the measured values.

#### Calculating plant photosynthesis and assimilate translocation

 Plant daily net photosynthesis (inducing photosynthesis of upright shoots and bent shoots) was calculated for the three treatments throughout the growth cycle. Photosynthesis was calculated from the first day after the start of treatments until the day of destructive measurements of upright shoots (in total 35 d). The hourly light intensity inside the greenhouse during the light period was used to calculate the hourly net photosynthetic rate of individual leaves. Daily photosynthesis of individual leaves was then calculated by summing up their hourly photosynthesis during the light period in each day. Daily photosynthesis of bent shoots and upright shoots was calculated as the sum of the daily photosynthesis of individual leaves attached on bent shoots and upright shoots. Daily whole-plant photosynthesis was then calculated as the sum of daily photosynthesis of bent shoots and upright shoots.

The fraction of assimilate translocation from bent shoots to upright shoots was approximated by the following approach. First the fraction of dry weight increase in upright shoots (*f*_d.wt_) due to the presence of bent shoots was calculated using eqn (5):

$$\begin{array}{l} f_{d.wt}=\left(Upright_{d.wt}-Upright_{d.wt,0B}\right)/Upright_{d.wt,0B} \end{array}$$ (5)

where Upright_d.wt_ is the upright shoot dry weight (g per plant) of 1B or 3B plants; and Upright_d.wt,0B_ is the upright shoot dry weight (g per plant) of 0B plants. Then, assuming that the fraction of cumulative assimilate increase in upright shoots is the same as the fraction of dry weight increase (*f*_d.wt_), the amount of assimilates accumulated in upright shoots in plants with a bent canopy was calculated using eqn (6):

$$\begin{array}{l} Upright_{ass}=\left(1+f_{d.wt}\right)\times Upright_{ass,0B} \end{array}$$ (6)

where Upright_ass_ is the amount of assimilates (mol per plant) accumulated in 1B or 3B upright shoots over the growth cycle; and Upright_ass,0B_ is the amount of assimilates (mol per plant) accumulated in 0B upright shoots, which is equal to the cumulative net photosynthesis over the growth cycle simulated by the 3-D model. Finally, the fraction of assimilates translocated from bent shoots to upright shoots (*f*_trans_) was calculated using eqn (7):

$$\begin{array}{l} f_{trans}=Upright_{ass}-Upright_{photo})/Bent_{photo} \end{array}$$ (7)

where Upright_photo_ and Bent_photo_ are the simulated net photosynthesis (mol per plant) by, respectively, upright shoots and bent shoots accumulated over the growth cycle.

Two additional simulations were performed. First, in the commercial greenhouse, leaf area index (LAI, m^2^ leaf m^–2^ floor) of bent shoots can be higher than in our experiment. Thus we further calculated daily photosynthesis for the 3B treatment using an LAI of 5 for bent shoots. The calculation was done for the whole growth period (35 d) according to the approach described previously. Secondly, a common situation in practice is that the rose flower shoots at different developmental stages are coexisting on the plants all year round, with harvestable shoots being pruned every day. Thus we further calculated daily photosynthesis for the 3B treatment with upright shoots consisting of shoots at different developmental stages. The phenotypes of individual upright shoots on the plants were randomly obtained from the non-destructive measurements (on days 6, 9, 14, 19 and 25) in the 3B treatment, resulting in mixed developmental stages of upright shoots on the same plant. In this simulation, plant net photosynthetic rate was calculated once, with the incoming light intensity being kept at the average level during the experiment (= 360 μmol m^-2^ s^-1^). The number of upright shoots was kept at 4, 6 and 8 shoots per plant, respectively, in the calculation, and bent shoot LAI was kept at the same level of the 3B treatment (= 3.6).

## RESULTS

### Effects of bent shoots on upright shoot morphology and biomass

**Fig. 1.**
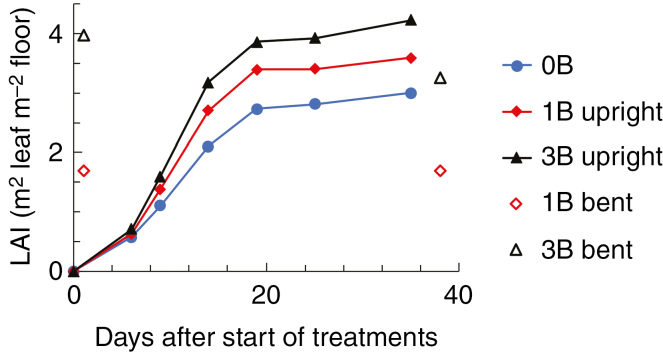
Leaf area index (LAI) of upright shoots during the experiment (filled symbols and solid lines) and LAI of bent shoots at the start and end of the experiment (open symbols). 0B, 1B and 3B represent no, one or three bent shoots per plant.

Plants with bent shoots (1B and 3B plants) had longer and thicker upright shoots than plants without bent shoots (0B plants) (Table 1). Stem biomass per unit of length was also higher in 1B and 3B upright shoots than in 0B upright shoots (Table 1), indicating that plants with bent shoots had stronger flower stems than plants without bent shoots. Individual internode length, mostly for internodes located in the middle of upright shoots, and individual internode diameter at harvest were increased when the number of bent shoots increased (Supplementary data Fig. S5A, B). Bent shoots did not affect the number of leaves on upright shoots (Table 1), but increased shoot total leaf area by increasing areas of individual leaves located in the middle of upright shoots (Table 1; Supplementary data Fig. S5C). This resulted in higher upright shoot LAIs of plants with bent shoots (Fig. 1). Leaf mass per area was not affected by bent shoots (Table 1), nor was leaflet number of individual leaves (Supplementary data Fig. S5D). Flower width at harvest was not affected by bent shoots (Table 1).

**Table 1.** Architectural traits, fresh weight and organ dry weight of individual upright shoots at harvest (35 d after the start of treatment)

Number of bent shoots per plant	0	1	3	s.e.m.
Stem length (cm)	76^b^	85^a^	88^a^	1.82
Stem diameter (mm)	6.09^c^	7.03^b^	7.67^a^	0.19
Stem biomass per unit of length (g cm^–1^)	0.063^b^	0.084^a^	0.101^a^	0.01
Leaf number	14^a^	14^a^	14^a^	0.44
Shoot leaf area (cm^2^)	1000^c^	1198^b^	1412^a^	44.10
Leaf mass per unit of area (g m^–2^)	41.4^a^	43.6^a^	42.9^a^	2.46
Flower width (cm)	11.9^a^	12.5^a^	12.6^a^	6.57
Shoot fresh weight (g)	58^b^	74^a^	85^a^	4.73
Shoot dry weight (g)	12.4^b^	16.7^a^	19.7^a^	1.38
Stem dry weight (g)	4.8^c^	7.2^b^	8.9^a^	0.61
Leaf dry weight (g)	4.1^b^	5.2^a^	6.1^a^	0.43
Flower dry weight (g)	3.5^b^	4.3^ab^	4.8^a^	0.37
Fraction of shoot dry weight allocated to stem	0.38^c^	0.43^b^	0.45^a^	0.01
Fraction of shoot dry weight allocated to leaf	0.33^a^	0.31^b^	0.31^b^	0.00
Fraction of shoot dry weight allocated to flower	0.28^a^	0.26^b^	0.24^b^	0.01

Letters following the numbers in each row indicate significant differences when comparing between treatments (*P* < 0.05). Stem diameter is the average diameter of all internodes on a shoot. Data were based on 18 replicate plants distributed over three blocks (*n* = 3).

Upright shoot fresh weight was 28 % higher in 1B and 47 % higher in 3B than in 0B plants (Table 1). Upright shoot dry weight was higher in 1B and 3B plants than in 0B plants, as was individual organ (stem, leaf and flower) dry weight of upright shoots (Table 1). The fraction of biomass allocated to the stem was higher in 1B and 3B upright shoots than in 0B shoots, which was at the expense of the fractions of biomass allocated to leaf and flower (Table 1).

### Effects of bent shoots on leaf photosynthetic parameters

Leaf photosynthetic parameters of upright shoots, including the maximum leaf photosynthetic rate (*A*_max_), dark respiration rate (*R*_d_) and quantum efficiency [*Φ*_CO2LL(inc)_], were hardly affected by the presence of bent shoots, except that *A*_max_ of lower leaves was higher in 0B upright shoots than in 1B and 3B upright shoots (Table 2). The number of bent shoots hardly affected photosynthetic parameters of leaves in bent shoots (Table 2). The distribution of *A*_max_ for leaves in upright shoots was correlated with the relative light intensity experienced by that leaf [*Q*/*Q*_0_ in eqns (2) and (3)]. By curve fitting eqn (3), leaf photosynthetic capacity of the most illuminated leaf in upright shoots (*A*_0,upright_) was quantified as 21.0 μmol CO_2_ m^–2^ s^–1^ and the slope *k* of eqn (3) [the same as the curvature factor *k* of eqn (2)] was determined to be 0.09 (Supplementary data Fig. S6). Assuming that the same correlation between *A*_max_ and *Q*/*Q*_0_ holds for leaves in bent shoots (i.e. the same *k* value for bent shoots), leaf photosynthetic capacity of the most illuminated leaf in bent shoots (*A*_0,bent_) was quantified as 16.9 μmol CO_2_ m^–2^ s^–1^ (Supplementary data Fig. S6), indicating that bending of a shoot decreased the photosynthetic capacity of its leaves.

**Table 2.** Leaf photosynthetic parameters estimated for leaves at the upper, middle and lower layers in upright shoots and for leaves of bent shoots

Number of bent shoots per plant	0	1	3	s.e.m.
*A*_max_ (μmol m^–2^ s^–1^)				
Upper leaf	20.2^a^	19.5^a^	19.7^a^	1.41
Middle leaf	18.9^a^	17.8^a^	18.5^a^	1.72
Lower leaf	16.8^a^	14.1^b^	12.5^b^	1.02
Leaf of bent shoots	–	15.7^a^	14.9^a^	0.79
*R*_d_ (μmol m^–2^ s^-1^)				
Upper leaf	1.25^a^	0.93^a^	0.90^a^	0.34
Middle leaf	0.75^a^	1.05^a^	1.05^a^	0.39
Lower leaf	0.64^a^	0.54^a^	0.45^a^	0.30
Leaf of bent shoots	–	0.42^a^	0.52^a^	0.12
*Φ*_CO2LL(inc)_ (mol CO_2_ mol^–1^ photon)				
Upper leaf	0.057^a^	0.055^a^	0.059^a^	0.00
Middle leaf	0.059^a^	0.059^a^	0.062^a^	0.00
Lower leaf	0.058^a^	0.057^ab^	0.051^b^	0.00
Leaf of bent shoots	–	0.053^a^	0.055^a^	0.00

Letters following the numbers in each row indicate significant differences when comparing between treatments (*P* < 0.05). Data were based on 6 replicate plants distributed over three blocks (n = 3).

### Calculations of daily plant net photosynthesis during the whole growth period

**Fig. 2.**
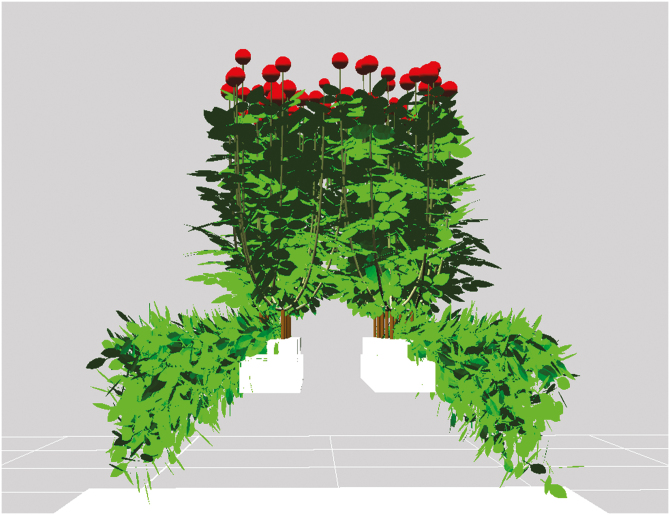
Three-dimensional representation of rose plants with three bent shoots per plant at the flowering stage simulated by the model.

The FSP model sufficiently captured the fraction of light interception in both upright shoots and bent shoots, with the *r*^2^ and rRMSE between the measured and simulated relative light intensities (*Q*/*Q*_0_) of 0.84 and 0.31, respectively (Supplementary data Fig. S7). This indicates that the 3-D representation in the model sufficiently represented the rose plants (both with and without bent shoots) for simulations of light absorption and photosynthesis (Fig. 2; Supplementary data S2). The model overestimated light levels below bent shoots in the 1B treatment (Supplementary data Fig. S7). This was possibly because in reality the gutters and construction bricks also blocked light, whereas these objects were not simulated in the model (Supplementary data Fig. S2), thus leading to an overestimation of light below bent shoots. The potential contribution to plant photosynthesis of the fraction of the limited amount of light reaching the ground (approx. 20 % of the incoming light; Supplementary data Fig. S7) reflected back upwards as simulated by the model was negligible (Supplementary data Fig. S8). Thus an overestimation of light below bent shoots in the 1B treatment did not affect the simulated plant photosynthesis. The simulated light level below bent shoots in the 3B treatment was similar to the measured light level, because high bent shoot LAI in 3B had blocked almost all the light.

**Fig. 3.**
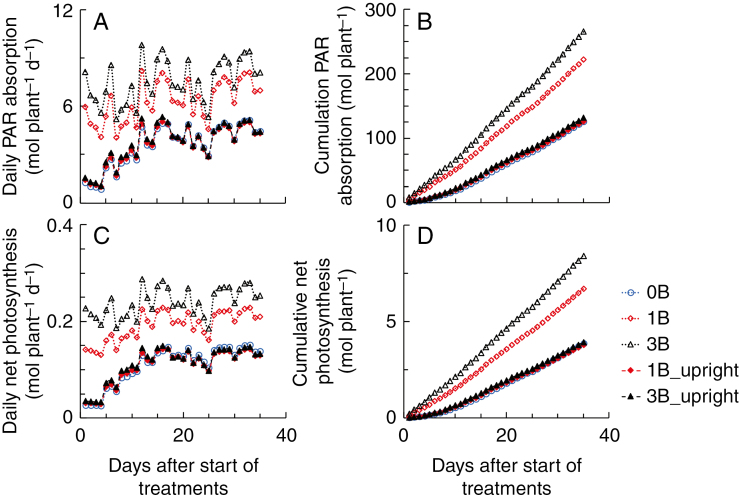
Simulated daily (A, C) and cumulative (B, D) photosynthetically active radiation (PAR) absorption (A, B) and net photosynthesis (C, D) of upright shoots (filled symbols with dashed lines) and the whole plant (open symbols with dotted lines). 0B, 1B and 3B represent no, one or three bent shoots per plant. PAR absorption and photosynthesis of upright shoots are equal to that of the whole plant in the 0B treatment.

Simulated PAR absorption and net photosynthesis of the upright shoots were hardly different between plants with and without bent shoots during the whole growth period (Fig. 3). When upright shoots were at their early developmental stages (on average five leaves appeared on a shoot), daily PAR absorption of the whole plant was three and five times higher, respectively, in 1B and 3B plants than in 0B plants (Fig. 3A), due to the additional leaf area of bent shoots. This resulted in daily photosynthesis being four and seven times higher, respectively, in 1B and 3B plants than in 0B plants (Fig. 3C). When upright shoots were fully developed, plant daily PAR absorption was 57 and 82 % higher, respectively, in 1B and 3B plants than in 0B plants (Fig. 3A). This resulted in plant photosynthesis being 51 and 83 % higher, respectively, in 1B and 3B plants than in 0B plants (Fig. 3C). At harvest, the cumulative plant PAR absorption was 76 and 111 % higher, and cumulative plant photosynthesis was 73 and 117 % higher, respectively, in 1B and 3B plants than in 0B plants (Fig. 3B, D). The cumulative PAR absorption by bent shoots in 3B plants was 40 % higher than that in 1B plants, resulting in 54 % higher bent shoot photosynthesis (Fig. 3B, D). At early stages of upright shoots, the relative contribution of bent shoots to daily PAR absorption of the whole plant was 76 % in 1B plants and 81 % in 3B plants (Supplementary data Fig. S9A). This results in the relative contribution of bent shoots to plant photosynthesis being 78 % in 1B plants and 85 % in 3B plants (Supplementary data Fig. S9C). With the growing of upright shoots, the relative contributions of bent shoots to daily plant PAR absorption and photosynthesis decreased (Supplementary data Fig. S9A, C). At harvest, bent shoots contributed 43 % cumulative PAR absorption and photosynthesis of 1B plants, and 51 % PAR absorption and 53 % photosynthesis of 3B plants (Supplementary data Fig. S9B, D).

**Fig. 4.**
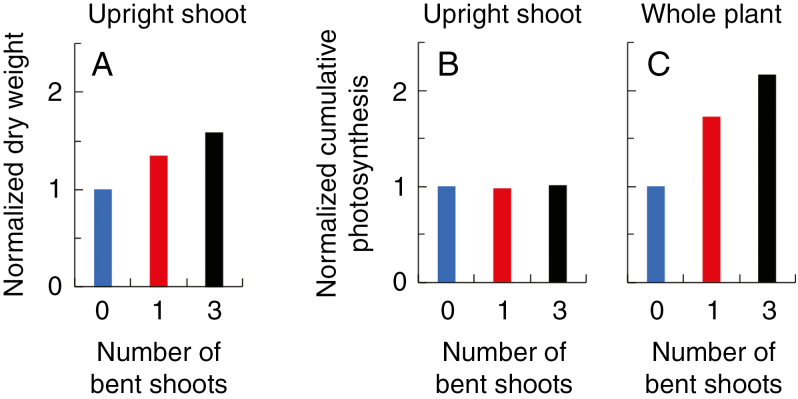
Upright shoot dry weight at harvest (A). Cumulative upright shoot (B) and plant (C) photosynthesis from bud break until harvest. All data were calculated relative to the data for plants with zero bent shoots.

To compare the differences of upright shoot photosynthesis and biomass production between treatments, we normalized these values in all treatments to values obtained in 0B (resulting in all values in 0B = 1). Upright shoot dry weight at harvest was 35 % higher in 1B and 59 % higher in 3B than in 0B plants (Fig. 4A). However, cumulative upright shoot photosynthesis at harvest was not different between treatments (Fig. 4B), indicating that the higher dry weight in 1B and 3B upright shoots did not come from photosynthesis of upright shoots themselves, and thus should come from their bent shoots. In addition, owning to the contribution of bent shoots, cumulative whole-plant photosynthesis at harvest was 73 % higher in 1B and was 117 % higher in 3B than in 0B plants (Fig. 4C). In both 1B and 3B plants, the fraction of increase in plant photosynthesis was higher than the fraction of increase in upright shoot dry weight (Fig. 4A, C). This indicates that bent shoots of 1B and 3B plants produced more assimilates than the amount that had been contributed to upright shoot dry weight increase.

**Fig. 5.**
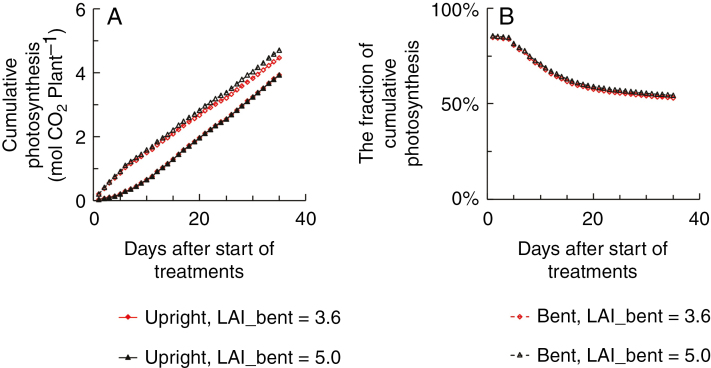
Simulated cumulative photosynthesis of upright shoots (filled symbols with solid lines) and bent shoots (open symbols with dashed lines) (A) and the cumulative photosynthesis by bent shoots as a fraction of the whole plant (B) in the treatment with three bent shoots per plant [3B treatment, with a leaf area index (LAI) of bent shoots being 3.6, in red] and in the simulation in which LAI of 3B plants increased to 5 (in black).

**Fig. 6.**
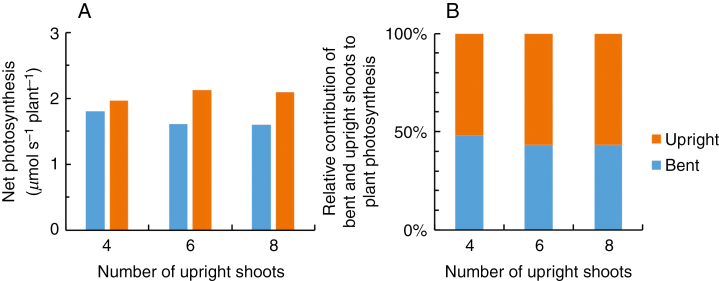
Simulated net photosynthesis (A) and the relative contribution to whole-plant photosynthesis (B) by bent shoots and upright shoots of 3B plants (in the treatment with three bent shoots per plant) consisting of 4, 6 or 8 upright shoots at different developmental stages.

As in the commercial rose greenhouse, LAI of bent shoots is often higher than in our experiment (bent shoot LAI in the experiment is given in Fig. 1), we calculated the effect of increasing bent shoot LAI to 5 on photosynthesis in 3B plants. Increasing bent shoot LAI led only to a 6 % increase in cumulative photosynthesis of bent shoots and a 3 % increase in cumulative plant photosynthesis at harvest when compared with an LAI of 3.6 in 3B plants in the experiment (Fig. 5A). The relative contribution of bent shoots to plant photosynthesis was slightly (2 %) higher at a bent shoot LAI of 5 than at an LAI of 3.6 (Fig. 5B). Furthermore, we tested if the contributions from the bent shoots would change if more upright shoots had been retained on the plant and if these shoots were in different developmental stages instead of all being in the same developmental stage. These scenarios are also common situations in the commercial greenhouse. When upright shoots consisted of shoots at different developmental stages, bent shoot photosynthesis decreased with the increasing number of upright shoots (Fig. 6A). In the presence of four upright shoots per plant, bent shoots contributed 48 % of plant photosynthesis, and this contribution decreased to 43 % in the presence of six and eight upright shoots per plant (Fig. 6B).

## DISCUSSION

### Bent shoot photosynthesis contributes to accumulation of upright shoot biomass

In cut-rose production, bent shoots are normally considered as an extra source of assimilates for the growth of upright flower shoots (Kim and Lieth, 2004). We found that upright shoot dry weight was increased by 35 % in plants with one bent shoot and by 59 % in plants with three bent shoots compared with plants with no bent shoots (Fig. 4A). The benefit of applying bent shoots in cut-rose production to increase upright shoot dry weight was also found by Kim and Lieth (2004). Previous studies attempted to use conventional canopy photosynthesis models (Pien *et al*., 2001; Kim and Lieth, 2002) and radioactively labelled carbon (Kajihara *et al*., 2009) to investigate the contribution of bent shoots in cut-rose production. Here, we quantified the contribution of bent shoots to whole-plant photosynthesis using a 3-D modelling approach. As far as we know, we are the first to quantify photosynthesis of different parts in the heterogeneous rose canopy using a 3-D model. The 3-D modelling approach presented here is also useful to assess the effect of canopy structure manipulations on plant photosynthesis and the associated crop production in various crops with other types of structure manipulations, such as leading branches along wires and pruning (Gladstone and Dokoozlian, 2003).

The increased upright shoot dry weight could come either from higher upright shoot photosynthesis in plants with bent shoots, or from assimilate translocation from bent shoots, or from both. Other parts of the plant (e.g. roots and the crown) were unlikely to be the causes of increased upright shoot dry weight, as plants both with and without bent shoots had similar potentials for upright shoots to import assimilates from these parts. Our model simulations clearly indicate that all the increased upright shoot dry weight was from additional photosynthesis by bent shoots, which may entail direct assimilate translocation to upright shoots, since net photosynthesis of upright shoots themselves was hardly affected by the presence of bent shoots. Mor and Halevy (1979) applied radioactively labelled carbon and showed that the growth of rose flower shoots, from axillary bud breaking until the appearance of the flower bud, is largely dependent on the supply of assimilates by the foliage of the previous growth cycles. Thus the additional photosynthesis by bent shoots may increase the assimilate supply during this period, resulting in an increase of assimilate translocation to upright shoots, a higher shoot dry weight and a higher LAI. At the flowering stage, the assimilates produced by bent shoots are mainly translocating to the roots and basal part of the plant (Kajihara *et al*. 2009).

Interestingly, the cumulative whole-plant photosynthesis was increased by 73–117 % in plants with bent shoots compared with plants without bent shoots (Fig. 4C). These fractions were higher than the fractions of increases in upright shoot dry weight in plants with bent shoots (Fig. 4A, C), indicating that bent shoots produced relatively more assimilates than the relative increase in upright shoot biomass. Based on our calculations [eqns (5–7)], the amount of increased assimilates in upright shoots accounted for 47–51 % of the cumulative assimilates produced by bent shoots during the growth period. However, to properly convert net photosynthesis to biomass growth, more costs need to be considered, such as the growth and maintenance respiration of organs other than leaves (root, stem and flower), leaf respiration during the night and the conversion cost from photosynthates to biomass. Thus, to support an increase in upright shoot biomass, the fraction of assimilates produced by bent shoots that are translocated to upright shoots should be higher than 47–51 %. Measured bent shoot dry weight at the end of treatments did not significantly differ from that at the start (Supplementary data Fig. S10), indicating that none of the assimilates produced by bent shoots remained in bent shoots. These assimilates may be used for plant respiration and the conversion cost between photosynthates and biomass, or they may be translocated to other parts (e.g. roots) of the plant (Kajihara *et al*., 2009).

Note that in the current model, direct light was not simulated; we only considered diffuse light. Sensitivity analysis showed that both total plant assimilation and total assimilation by bent shoots decreased with an increase in the fraction of direct light (Supplementary data Fig. S11A–H). This leads to the result that the fraction of bent shoot photosynthesis to whole-plant photosynthesis was hardly affected by the fraction of direct light that was considered in the light model (Supplementary data Fig. S11I–L). Therefore, the choice to use only diffuse light in our model analysis should not have had a significant impact on our conclusions. An additional model simplification was the absence of simulation of carbon allocation and growth. We did not include such a carbon allocation model, due to the absence of a reliable model capturing the distribution of carbon between bent and upright shoots. Radioactive carbon labelling studies already showed that at the flowering stage, a large fraction of the assimilates produced by bent shoots was translocated to roots and the crown (Kajihara *et al*., 2009). To model the export of assimilates produced by bent shoots in cut-rose production, further studies on the carbon allocation at different developmental stages of the flower shoots are needed, especially studies at the early developmental stage when photosynthesis by the flower shoots is not enough to support their own growth.

### Bent shoots increased morphological quality traits of upright shoots

The benefits of applying bent shoots in cut-rose production, e.g. increasing shoot fresh weight and stem length, has been demonstrated in previous studies (Warner and Erwin, 2002; Kim and Lieth, 2004) as well as in ours (Table 1). Longer stems and larger shoot leaf areas of upright shoots in plants with bent shoots were the result of longer internodes and larger leaves located in the middle of the shoot, whereas internode length and leaf area at lower and higher ranks of upright shoots were hardly affected by bent shoots (Supplementary data Fig. S5A, C). This is likely to be because at early developmental stages, sink demand is relatively low and upright shoots could use assimilates stored in the basal part of the plant, while at later developmental stages, upright shoots could produce enough assimilates by themselves (Baille *et al*., 2006), resulting in less pronounced effects of assimilate supply from bent shoots on the growth of organs which appeared at early or late developmental stages. However, diameters of all individual internodes in upright shoots were larger in plants with bent shoots than in plants without bent shoots (Supplementary data Fig. S5B), indicating that positive effects of assimilate supply from bent shoots on upright shoot organ size exist during the whole shoot growth period. This is in line with Marcelis-van Acker (1994) who found strong positive effects of assimilate supply on stem length and diameter and leaf area in rose. The effects of bent shoots on flower size and dry weight were less pronounced compared with the effects on the stem and leaf (Table 1), as also found by Kim and Lieth (2004).

Although upright shoot leaf area was increased by 20–40 % in plants with bent shoots compared with plants without bent shoots, this hardly affected upright shoot light absorption and photosynthesis (Table 1; Fig. 3). This may be caused by the fact that upright shoots in plants without bent shoots received additional light reflected from the ground. When bent shoots were present, this reflected light was mostly absorbed by the bent shoots rather than by the upright shoots. When we ran a simulation assuming that the ground does not reflect any light, upright shoots in plants that had bent shoots increased their light absorption by 6–11 % compared with upright shoots in plants without bent shoots (Supplementary data Fig. S12A). This led to an increase of upright shoot photosynthesis by 5–9 % (Supplementary data Fig. S12B). In the case of 100 % light reflection by the ground, upright shoot light absorption and photosynthesis were even lower in plants with bent shoots than in plants without bent shoots, because the latter received more light reflected from the ground (Supplementary data Fig. S12). However, even in the case of a zero ground reflectance, the increases of upright shoot light absorption (6–11 %) and photosynthesis (5–9 %) in plants with bent shoots were not proportional to the increase in upright shoot leaf area (20–40 %) (Table 1; Supplementary data Fig. S12). This may be caused by the fact that upright shoot LAI reached relatively high levels soon after the start of treatment (Fig. 1). Thus, a further increase in upright shoot LAI did not result in a proportional increase in light capture and photosynthesis by upright shoots. Note that due to the patchy distribution of leaves in a heterogeneous canopy (e.g. the rose canopy), the same LAI value could indicate much more dense leaves occupying part of the area compared with a homogeneous canopy. The LAI of bent shoots was found to be approx. 3.8 in practice (Warner and Erwin, 2002), which is similar to our treatment with three bent shoots per plant (Fig. 1). This level of LAI for bent shoots is possibly a reasonable level to keep in practice as our simulations showed that increasing LAI to a higher level (= 5) hardly increased photosynthesis of bent shoots and the whole plants (Fig. 5A). However, the optimal bent shoot LAI is also relevant to the season of the year and the LAI of upright shoots (Kim and Lieth, 2002). When it comes to the winter production season during which the ambient light level is relatively low, or when the production system has a higher upright shoot LAI than in our experiment, the optimal LAI of bent shoots should be lower than the level defined in our study.

### Calculating photosynthesis of a heterogeneous canopy

Previously, Baille *et al*. (2006) quantified the biomass import and export in rose flower shoots, under the assumption of a spatially uniform light environment around the growing flower shoots. This assumption, however, does not hold for a rose canopy with both vertically grown upright shoots and horizontally grown bent shoots, in which the light conditions can be quite heterogeneous. We represented the heterogeneous rose canopy in 3-D using an FSP modelling approach (Fig. 2), which allowed us to simulate light absorption and photosynthesis at the individual leaf level without assuming a homogeneous canopy. Using the FSP modelling approach, the light environment inside the greenhouse could also be simulated in more detail, by including the continuously changing position of the sun, lamps at different positions in the greenhouse and the greenhouse structure that causes the reduction of light (Buck-Sorlin *et al*., 2011). However, we did not simulate the light environment at such a detailed level, as this was not the focus of our study, and this also would have required a larger computational capability. As previously discussed, we carried out sensitivity analyses to test the effects of different sky dome light distributions on our simulation results, showing that our conclusions were not affected by the assumed distribution (Supplementary data Fig. S11).

It is worthwhile to note that the approach we used to derive the distribution of leaf photosynthetic capacity in upright shoots and bent shoots was originally proposed in homogeneous canopies. The principle behind this approach is largely based on the premise that light distribution in the canopy drives leaf nitrogen distribution, which in turn determines the distribution of leaf photosynthetic capacity in the canopy (Hirose and Werger, 1987; Hikosaka, 2014). However, some studies argue that plants could also potentially distribute their leaf nitrogen according to the gradient of the red to far-red ratio in the canopy (Pons *et al*., 1993; Pons and De Jong-Van Berkel, 2004). Our results indicate that this approach cannot be used for the entire heterogeneous rose canopy, since we found that *A*_max_ of leaves in bent shoots was apparently lower than *A*_max_ of leaves in upright shoots at the same relative light intensity (Supplementary data Fig. S6). However, we can use this approach to derive the distribution of leaf photosynthetic capacity in upright shoots and bent shoots separately, since the gradient of *A*_max_ was found in both upright shoots and bent shoots (González-Real and Baille, 2000; González-Real *et al*., 2007). The coefficient of the relationship between light distribution and *A*_max_ distribution [*k* in eqns (2) and (3)] in upright shoots of rose plants was determined as 0.09 (Supplementary data Fig. S6), which is lower than the average value (approx. 0.37) found across different species, but is still within the range of woody species (Hikosaka *et al*., 2016a). We assumed the same *k* also holds for bent shoots (Supplementary data Fig. S6), based on the fact that extinction coefficients of leaf nitrogen distribution in upright shoots and in bent shoots are similar (González-Real *et al*., 2007). In fact, sensitivity analysis showed that the *k* value hardly affected bent shoot photosynthesis (Supplementary data Fig. S13), possibly because (1) the incoming light intensity for bent shoots was at a relatively low level and, in such a case, photosynthesis was not much affected by *A*_max_, and (2) the light gradient across bent shoots was not very large. However, *A*_max_ of the most illuminated leaf in upright shoots (*A*_0,upright_) was higher than in bent shoots (*A*_0,bent_) (Supplementary data Fig. S6), indicating that shoot bending may decrease photosynthetic capacity of leaves in bent shoots, which is also found by others and in other crops (Schubert *et al*., 1995; Kim *et al*., 2004).

Several explanations are proposed to explain the lower photosynthesis of leaves in bent shoots. Kim *et al*. (2004) found that the xylem tissue of rose bent shoots was damaged due to bending, and this could reduce hydraulic conductivity of bent shoots and decrease photosynthesis. The damage of xylem tissues, however, could recover over time under the possible involvement of the phytohormone ethylene (Mitchell, 1996; Liu and Chang, 2011). Even when xylem conductivity is not affected by shoot bending, it induces a transient variation in the hydraulic pressure within the xylem of bent shoots (Lopez *et al*., 2014). This transient increase in the xylem pressure could be rapidly propagated along the vascular system and such hydraulic signals could be converted into the chemical signal abiscisic acid (ABA), which is relevant to stomatal closure in leaves (Christmann *et al*., 2013; Huber and Bauerle, 2016). In addition, a decrease in Rubisco is found in leaves of downward bending shoots of grapevine (Schubert *et al*., 1995). Given that there is no consensus on the mechanism of the effect of shoot bending on photosynthesis of its leaves, while shoot bending is a common practice used in woody crops (e.g. rose) and fruit trees (e.g. pear) (Schubert *et al*., 1995; Ito *et al*., 1999; Kim *et al*., 2004; Liu and Chang, 2011), further studies on the mechanisms are worthwhile.

### Conclusions

Bent shoots increased upright shoot fresh and dry weight, and improved shoot morphological quality (e.g. longer and thicker stems). The presence of bent shoots increased plant photosynthesis by 73–117 %, whereas it did not affect photosynthesis of upright shoots. Therefore, model simulations of photosynthesis revealed that the increased upright shoot dry weight (by 35–59 %) in plants with bent shoots resulted entirely from the contribution of additional photosynthesis by bent shoots, as this was the only assimilate source that could induce differences in upright shoot growth apart from their own photosynthesis. At least 47–51 % of the assimilates produced by bent shoots were translocated to upright shoots to support their dry weight increase. The remaining assimilates, however, did not remain in bent shoots, but may be used for maintenance respiration and energy cost during the conversion of photosynthates to biomass, or may be translocated to other parts (e.g. roots) of the plant. Based on our simulations, we conclude that in cut-rose production, the increased flower shoot dry weight and quality can be attributed almost entirely to the assimilate supply from bent shoots. Functional–structural plant models can be very useful to quantify the relative contributions of upright shoots and bent shoots to photosynthesis of the heterogeneous rose canopy, and thus to create a balance between the number of harvestable flower shoots and shoot quality. Moreover, FSP models are useful tools to assess the effects of other types of canopy structure manipulations (e.g. leading and pruning) on plant photosynthesis and the associated crop production.

## SUPPLEMENTARY DATA

Supplementary data are available online at https://academic.oup.com/aob and consist of the following.

Figure S1: pictures of different developmental stages of the rose plants in the experiment.

Figure S2: comparisons of real rose plants and simulated rose plants.

Figure S3: different types of angle distribution for leaves of bent shoots simulated by the model.

Figure S4: the effect of bent shoot leaf angle distribution on light absorption of bent shoots.

Figure S5: measurements of internode length and diameter, leaf area and leaflet number.

Figure S6: the relationship between leaf photosynthetic capacity and the relative light intensity experienced by the leaf.

Figure S7: comparison between measured and simulated relative light intensity.

Figure S8: the effect of the ground light reflectance on the simulated light absorption and photosynthesis of bent shoots as a fraction of the whole plant.

Figure S9: simulated daily and cumulative light absorption and photosynthesis by bent shoots as a fraction of the whole plant.

Figure S10: bent shoot dry weight measured at the start and end of the experiment.

Figure S11: the effect of the fraction of direct light on total plant assimilation, total assimilation by bent shoots and bent shoot photosynthesis as a fraction of the whole plant.

Figure S12: the effect of light reflectance of the ground on light absorption and photosynthesis of upright shoots.

Figure S13: sensitivity analysis of the effect of model parameter *k* on photosynthesis of upright shoots and bent shoots.

Method S1: the methodology of estimating leaf photosynthetic parameters.

mcz150_suppl_Supplementary_Materials

## FUNDING

This work was supported by the China Scholarship Council (CSC) [grant no 201406850003] and the project ‘More roses for less’ [grant no. 870.15.040] funded by the Netherlands Organisation for Scientific Research (NWO), Signify and Glastuinbouw Nederland.
