# Supplementary material for: Quantifying the contribution of bent shoots to plant photosynthesis and biomass production of flower shoots in rose (Rosa hybrida) using a functional–structural plant model
Source: Ann Bot. 2019 Sep 24;126(4):587–99. doi: 10.1093/aob/mcz150 (PMC7489084; doi:10.1093/aob/mcz150)
Supplement: mcz150_suppl_Supplementary_Materials [file mcz150_suppl_supplementary_materials.docx]

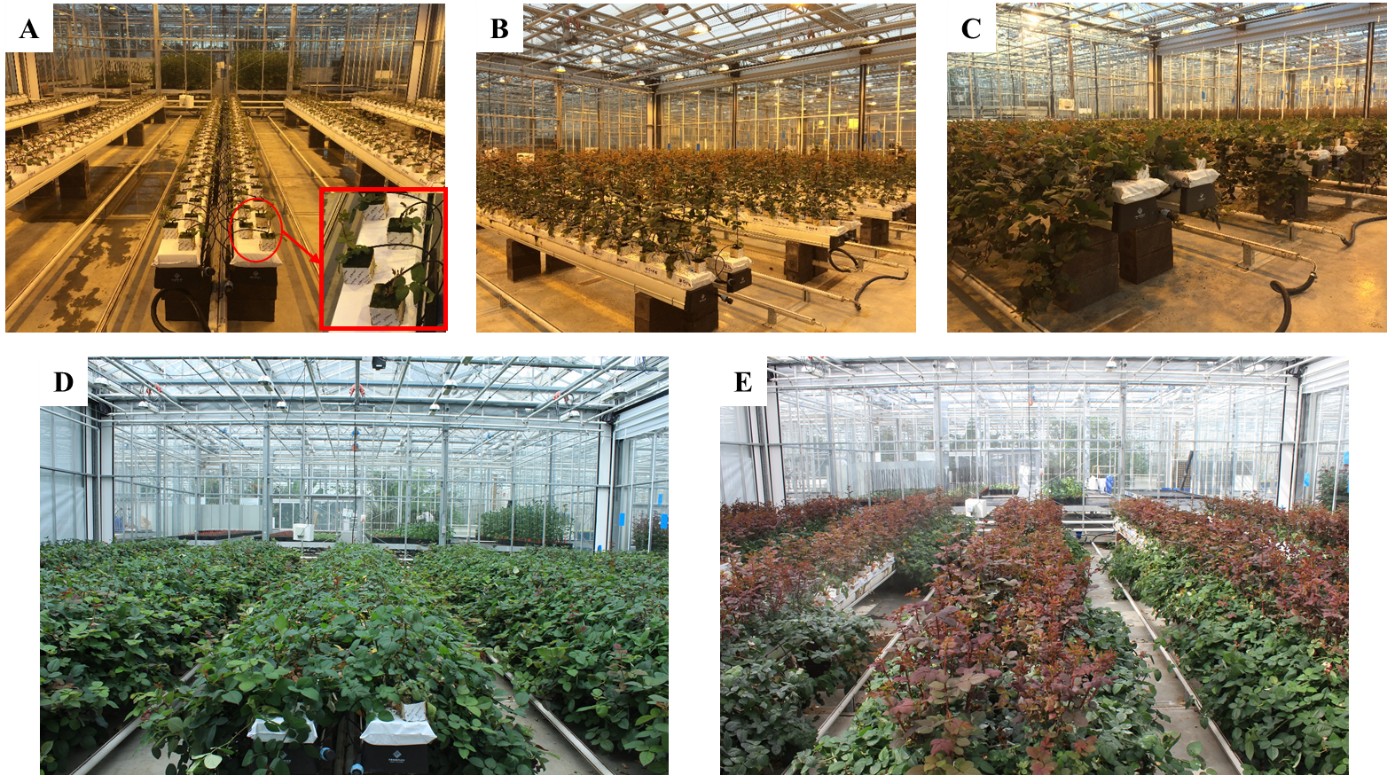


Fig. S1. Pictures of different developmental stages of the rose plants in the experiment. (A) Rooted rose-cuttings bearing a shoot (the primary shoot) at the start of the experiment. (B) All primary shoots had developed a flower bud. (C) All primary shoots were bent downwards. (D) All secondary shoots were bent downwards, and the plants were pruned to have two cuttings on each plant. (E) On average four axillary buds per plant were sprouted and treatments started; part of the bent shoots were missing in this picture due to the removal of bent shoots in that treatment.


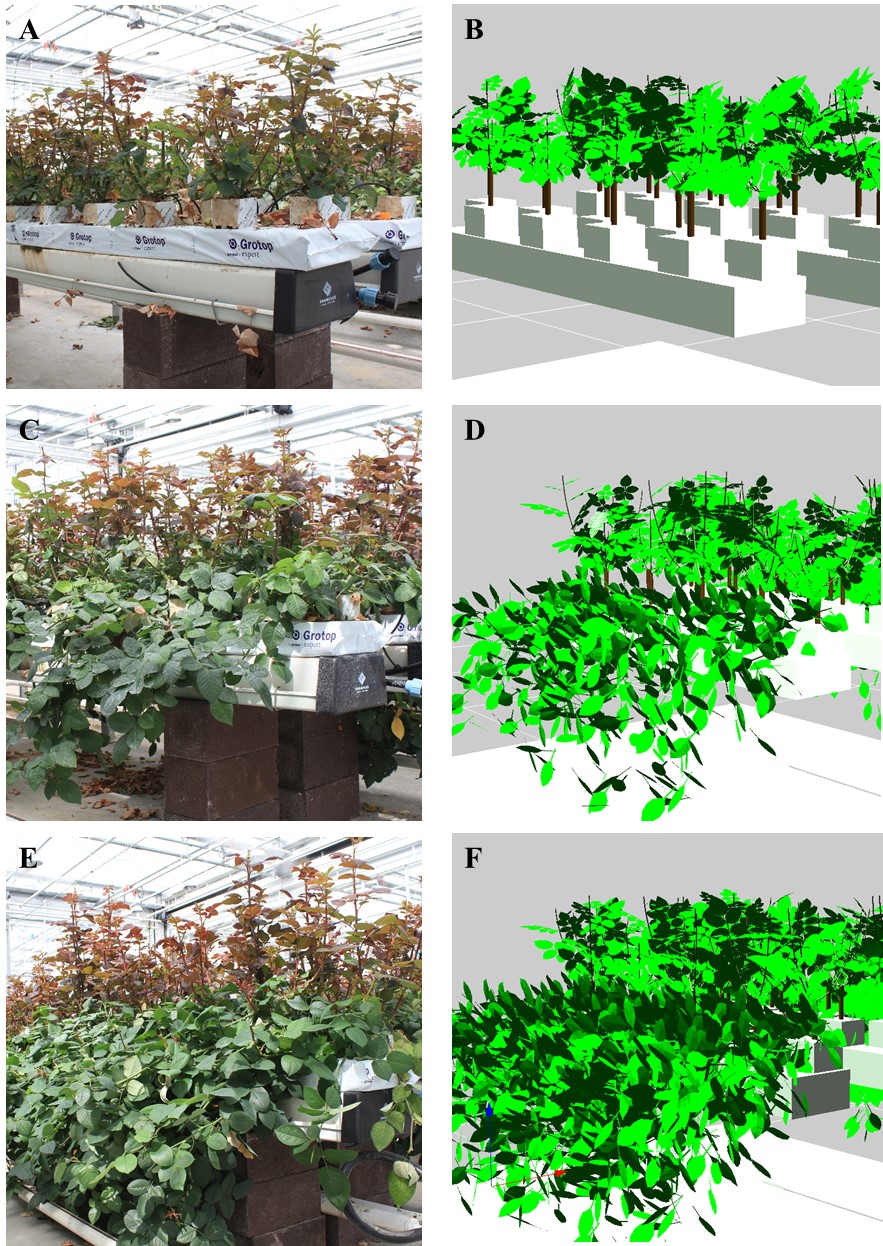


Fig. S2. Comparisons of real rose plants (A,C,E) and simulated rose plants (B,D,F) with 0 (A,B), 1 (C,D) and 3 (E,F) bent shoots per plant at early developmental stage (on average 5 leaves appeared on each upright shoot).


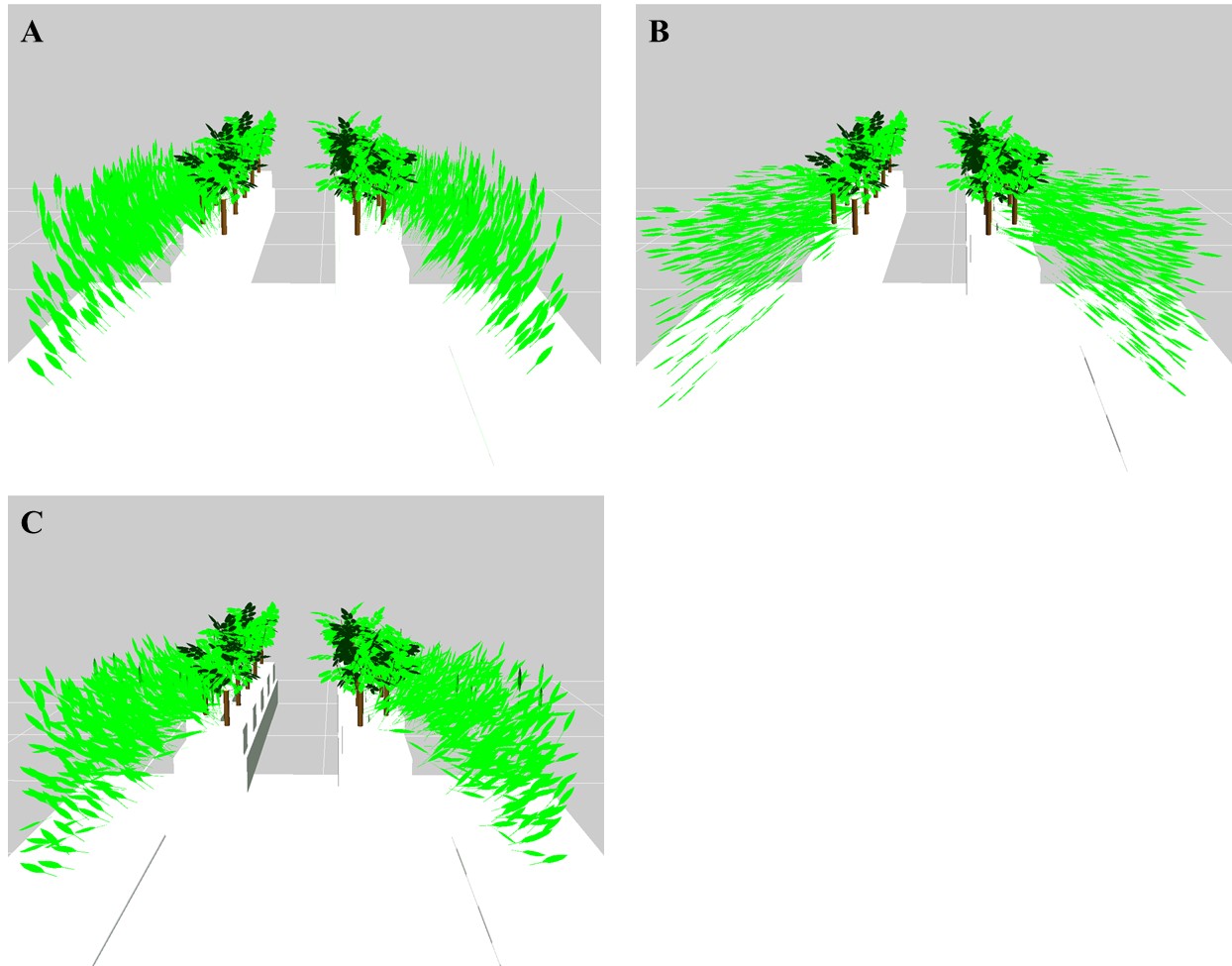


Fig. S3. An example of different types of angle distributions for leaves of bent shoots in treatment with one bent shoot per plant, at early developmental stage of upright shoots. (A) All leaves are vertical to the direction of the stem. (B) All leaves are horizontal to the direction of the stem. (C) Random distribution of leaf angles.


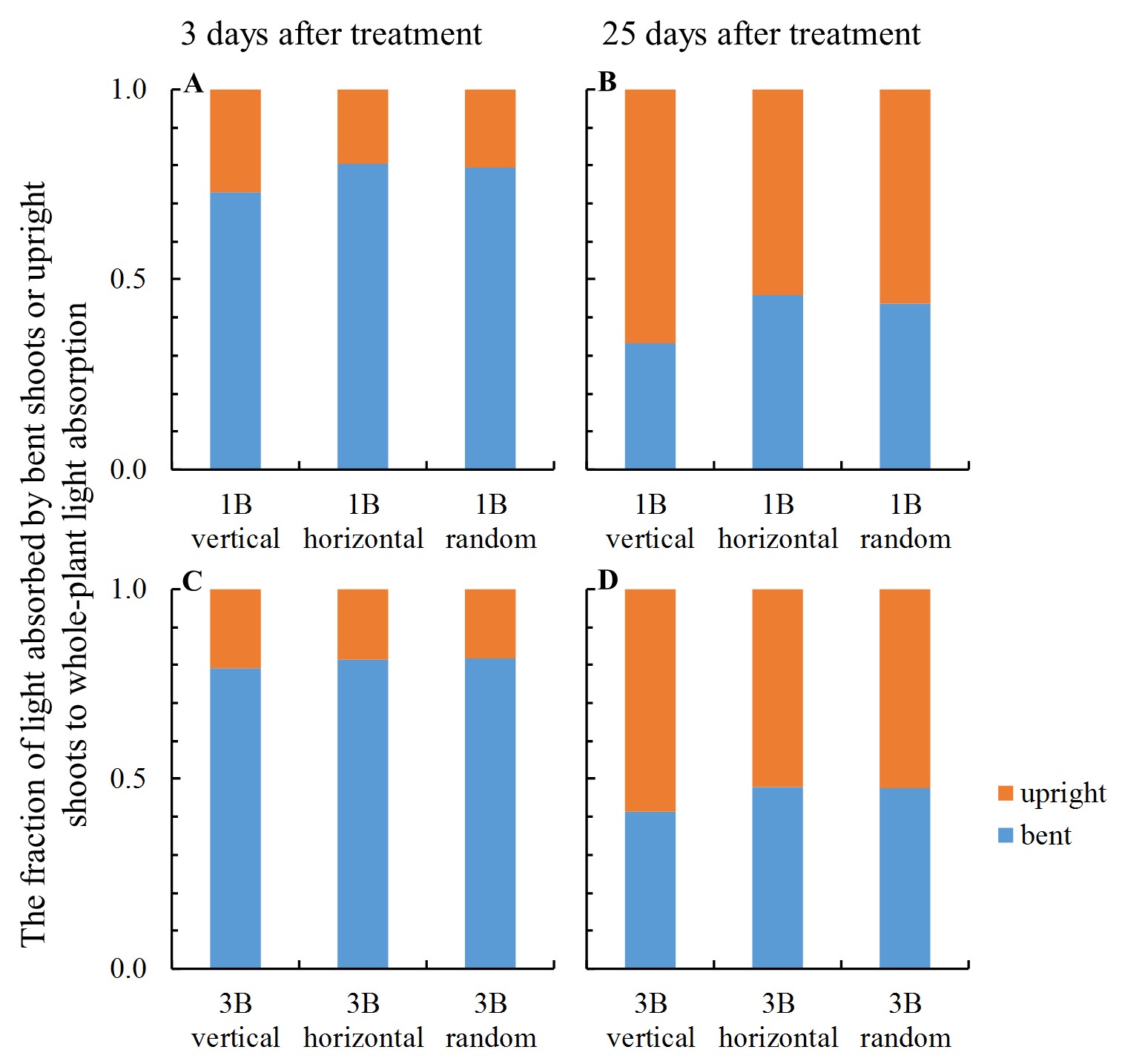


Fig. S4. The effect of leaf angle distribution on the fraction of light absorbed by bent shoots to whole-plant light absorption for treatment with one bent shoot per plant (A,B) and treatment with three bent shoots per plant (C,D), at early (A,C) and late (B,D) developmental stage of upright shoots. Illustrations of vertical, horizontal and random leaf angle distribution of bent shoots can be found in Fig. S3.


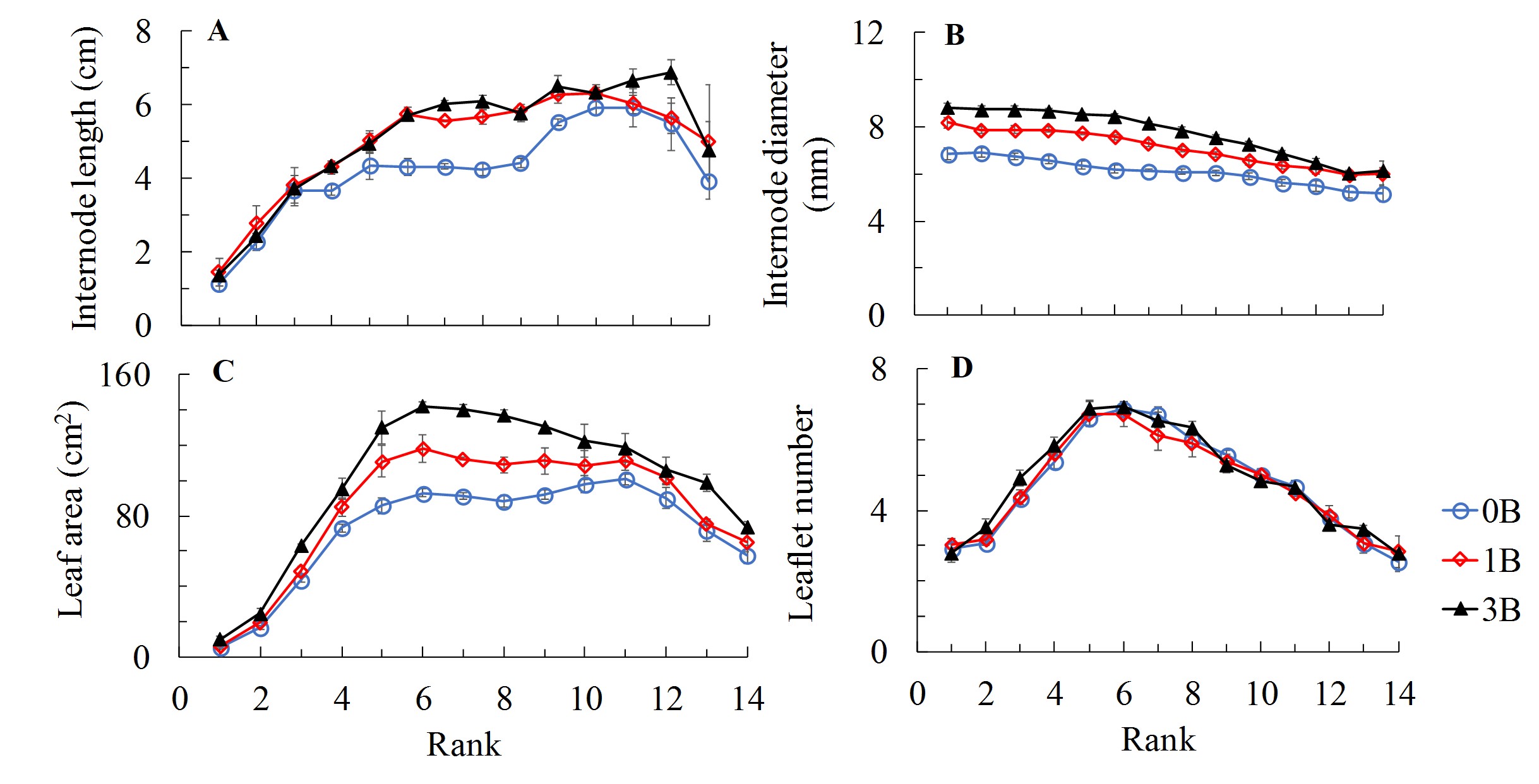


Fig. S5. Measurements of internode length (A), internode diameter (B), leaf area (C), and leaflet number (D) at harvest. Rank numbers are counted from the base towards the flower on the shoot. Error bars are standard errors of means. 0B, 1B and 3B represent no, one or three bent shoots per plant.


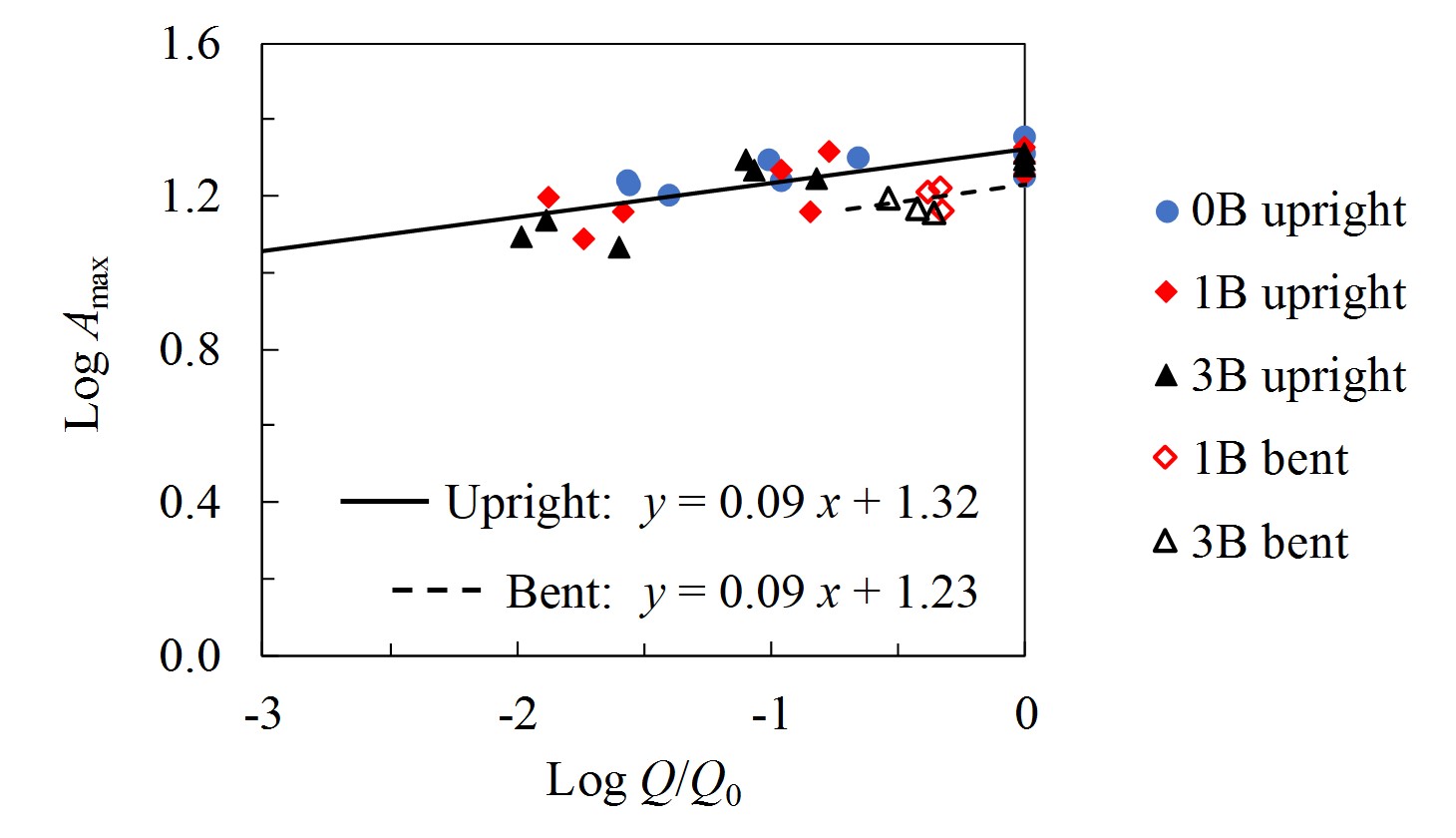


Fig. S6. The relationships between the logarithm of the maximum leaf photosynthetic rate (*A*_max_) of upper, middle and lower leaves in upright shoots and leaves in bent shoots and the logarithm of the relative light intensity (*Q*/*Q*_0_) experienced by that leaf (see Eq. 3: $\log A_{max}=k\times\log Q/{Q_{0}}+\log A_{0}$). Closed symbols are upright shoots. Open symbols are bent shoots. Solid line is the fitted curve for leaves in upright shoots in the three treatments. Dashed line is the fitted curve for leaves in bent shoots in 1B and 3B treatments, obtained by only fitting the intercept, but keeping the slope the same as for the upright shoots. Intercepts of the solid line and dashed line with *y* axis represent the logarithm of *A*_max_ of the most illuminated leaf in upright shoots (Log *A*_0,upright_) and in bent shoots (Log *A*_0,bent_) respectively. Slope of the solid line and dashed line represents the *k* value used for both upright shoots and bent shoots. 0B, 1B and 3B represent no, one or three bent shoots per plant.


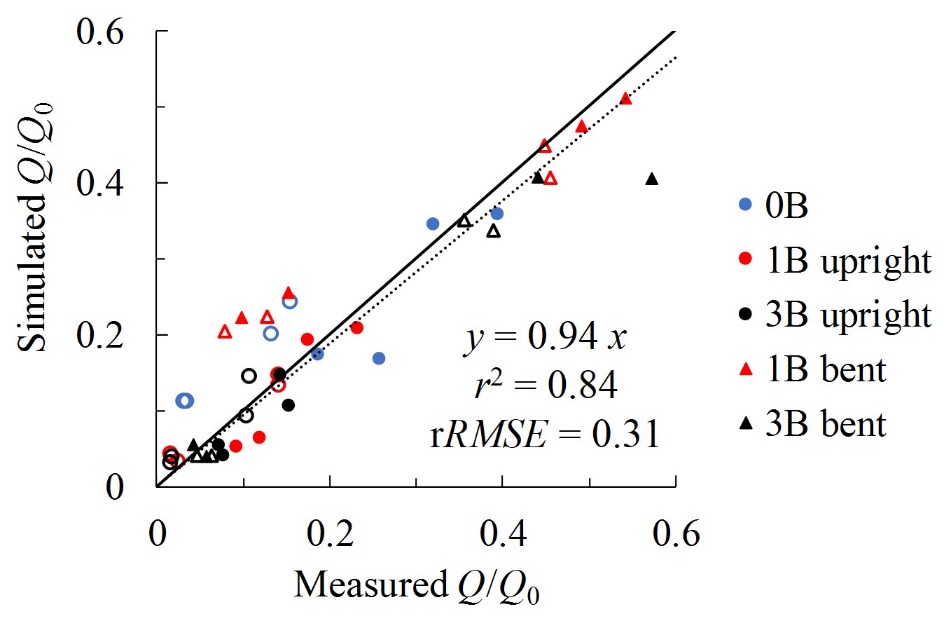


Fig. S7. Measured and simulated relative light intensities, i.e. *Q*/*Q*_0_, where *Q* is light intensity at middle or bottom of upright shoots (circles) or above or below bent shoots (triangles), and *Q*_0_ is light intensity above upright shoots. Measurements were conducted on day 13 (closed symbols) and 30 (open symbols) after the start of treatments. Solid line is the 1:1 line. Dotted line is the fitted curve for all data points by forcing the line goes through the origin. r*RMSE* is the relative root-mean-square error. 0B, 1B and 3B represent no, one or three bent shoots per plant.

Fig. S8. The effect of photosynthetically active radiation (PAR) reflection by the ground on the simulated light absorption and photosynthesis by bent shoots as a fraction of the whole plant. Simulations were performed for treatment with one bent shoot per plant. In the simulations, incoming light intensity was kept at the average value during the experiment (= 360 *μ*mol m^-2^ s^-1^). PAR reflectance of 0.3 is the value used in model validation (Fig. S7).


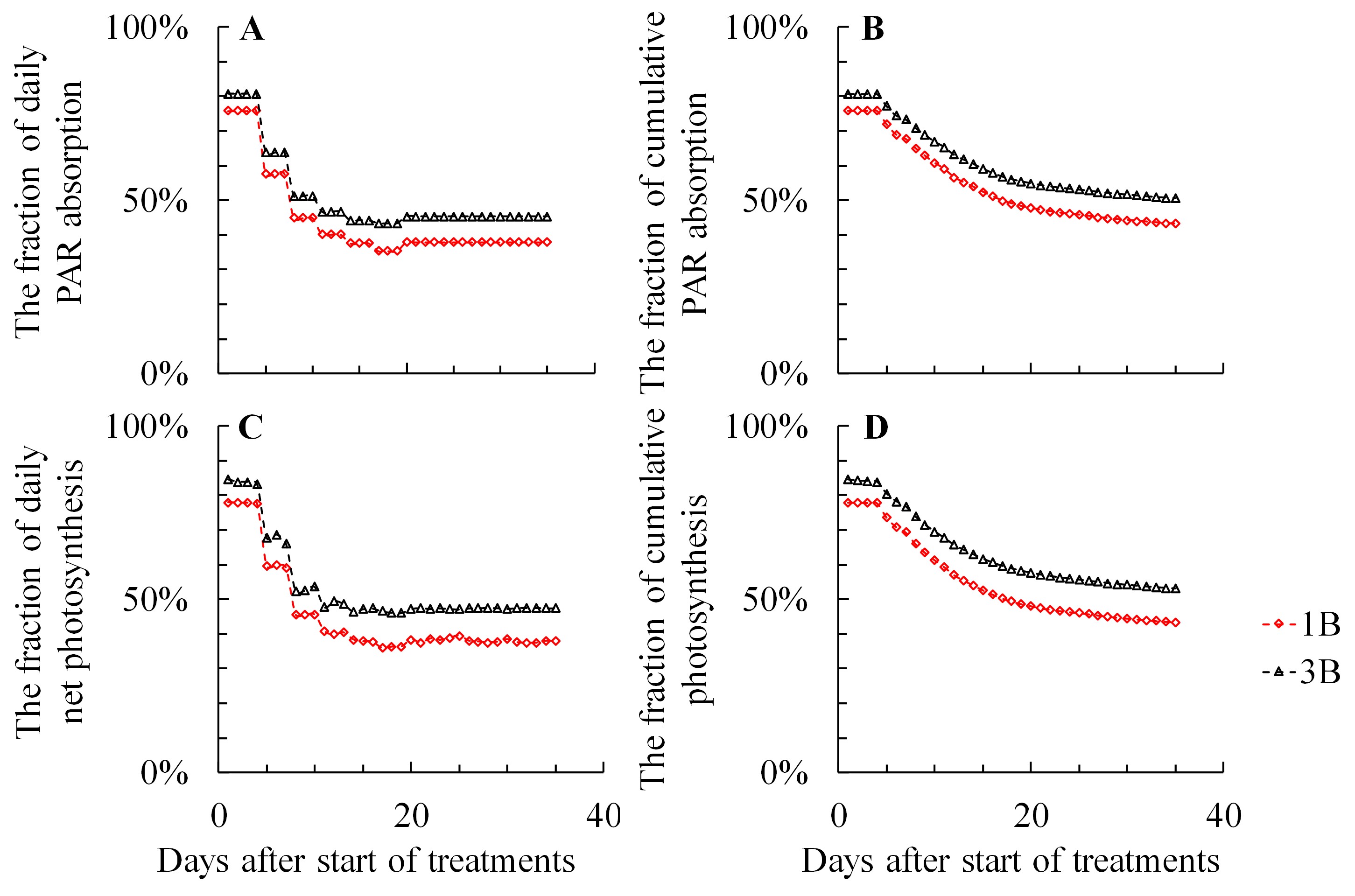
Fig. S9. Simulated daily (A,C) and cumulative (B,D) photosynthetically active radiation (PAR) absorption (A,B) and net photosynthesis (C,D) by bent shoots as a fraction of the whole plant. 1B and 3B represent respectively one and three bent shoots per plant.

Fig. S10. Individual bent shoot dry weight measured at the start and end of experiment. Letters above each bar indicate significant difference. Error bars are standard errors of means.

Fig. S11. Effects of the fraction of direct light on total plant assimilation (A-D), total assimilation by bent shoots (E-H) and on the fraction of bent shoot photosynthesis to whole-plant photosynthesis (I-L). Simulations were conducted at an incoming light intensity of 360 *μ*mol m^-2^ s^-1^ (average light level during the experiment) (A,B,E,F,I,J) or 1200 *μ*mol m^-2^ s^-1^ (the highest light level that plants experienced in the experiment) (C,D,G,H,K,L), with the direct light source located at a solar angle of 30° (A,C,E,G,I,K) or 90° (B,D,F,H,J,L). Dotted lines with open circles are simulation results using upright shoot architecture at 3 days after treatment. Solid lines with closed circles are simulation results using upright shoot architecture at 25 days after treatment.


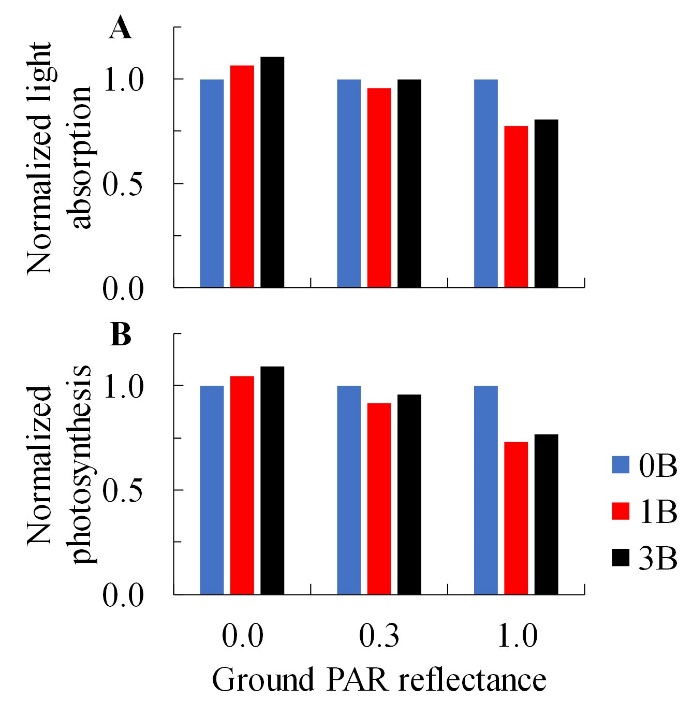


Fig. S12. Simulated light absorption (A) and photosynthesis (B) by upright shoots with the reflectance of photosynthetically active radiation (PAR) by the ground is 0.0, 0.3 (the value used in simulations presented in the main text) or 1.0. In the simulations, incoming light intensity was kept at the average value during the experiment (= 360 *μ*mol m^-2^ s^-1^). 0B, 1B and 3B represent no, one or three bent shoots per plant. All data were calculated relative to the data for 0B plants.


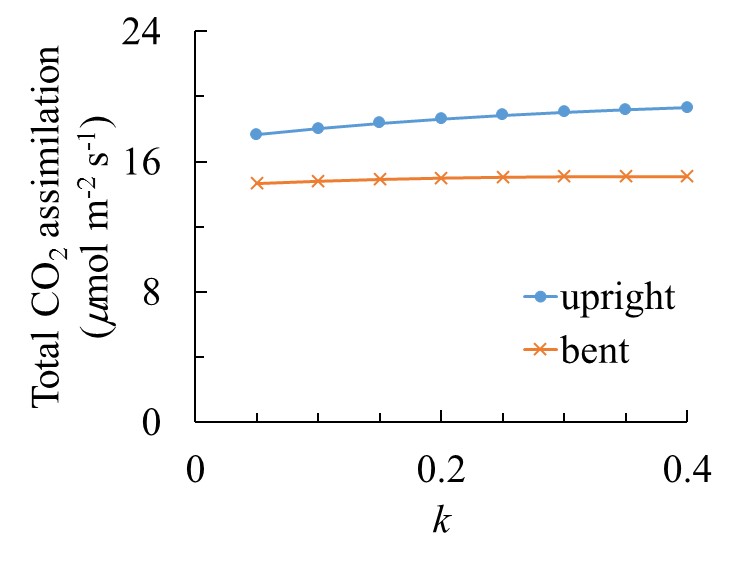


Fig. S13. Sensitivity analysis on the effect of *k* value (the coefficient describing the relationship between the gradients of light and leaf photosynthetic capacity in a canopy) on total CO_2_ assimilation by upright shoots and bent shoots. The sensitivity analysis was conducted for the treatment with three bent shoots per plant (3B), with the architecture of 3B upright shoots at their harvestable stage, and with an incoming light intensity of 360 *μ*mol m^-2^ s^-1^ which represented the average light level during the experiment inside the greenhouse.

Method S1. Estimating leaf photosynthetic parameters

The combined measurement of gas exchange and chlorophyll fluorescence was used to determine a set of photosynthetic parameters as reported in the main text, using the method as described by Yin et al (2009, 2014). Compared with conventional photosynthetic parameter estimations, this method explores the decrease of the operating photosystem II photochemical efficiency with increasing irradiance, which is likely more relevant to crops grown in relatively low light environment such as in the greenhouse.

The operating efficiency of photosystem II (PSII) photochemistry (*Φ*_2_) at each irradiance level was measured from fluorescence signals, according to Genty et al (1989), as:

$\text{}_{2}=1-{F_{s}}/{F_{m}^{'}}$ (Eq. S1)

where *F*_s_ is the steady-state fluorescence and *F*_m_’ is the maximum fluorescence. *F*_s_ and *F*_m_’ were obtained directly from the combined gas exchange and chlorophyll fluorescence measurements.

First, according to Yin et al (2009), the decrease of *Φ*_2_ with increasing irradiance can be fitted to the equation below:

$\text{}_{2}=(\text{α}_{2LL}I_{abs}+J_{2max}-\sqrt{\left( \text{α}_{2LL}I_{abs}+J_{2max} \right)^{2}-4\text{β}J_{2max}\text{α}_{2LL}I_{abs}})/(2\text{β}\text{}_{2}I_{abs})$ (Eq. S2)

in which $\rho_{2}={\alpha_{2LL}}/{\text{}_{2LL}}$ (Eq. S3)

$\alpha_{2LL}=\text{}_{2LL}\times(1-f_{cyc})/(\frac{\text{}_{2LL}}{\text{}_{1LL}}+\left( 1-f_{cyc} \right))$ (Eq. S4)

where *I*_abs_ is the irradiance absorbed by the leaf, which is calculated as *I*_inc_ multiplied by leaf absorbance; *J*_2max_ (*μ*mol m^-2^ s^-1^) is the total rate of electron transport passing PSII under saturating irradiance; *β* is the curvature factor; *ρ*_2_ is the factor of excitation partitioning to PSII; *α*_2LL_ (mol e^−^ mol^-1^ photon) is the PSII photochemical efficiency under strictly limiting light on the basis of light absorbed by both PSI and PSII; *Φ*_1LL_ (mol e^−^ mol^-1^ photon) is the photochemical efficiency of PSI and a value of 1.0 can be used for C_3_ species; *f*_cyc_ is the fraction of cyclic electron transport in the total electron flux passing PSI and a value of 0.05 can be used for C_3_ species (Yin et al. 2014). The estimated from *Φ*_2LL_ from this fitting does not depend on the pre-set values of *Φ*_1LL_ and *f*_cyc_.

Next, using the combined measurements of net rate of photosynthesis (*A*, mol CO_2_ m^-2^ s^-1^) and *Φ*_2_ at different incident irradiance levels (*I*_inc_, *μ*mol m^-2^ s^-1^), a lumped parameter *s*’ and the respiration rate in the light or called day respiration (*R*_d_, *μ*mol CO_2_ m^-2^ s^-1^) were estimated, based on the linear regression equation (Yin et al. 2014):

$A=s^{'}\left( \frac{I_{inc}\text{}_{2}}{4} \right)-R_{d}$ (Eq. S5)

Thirdly, according to Yin et al (2014), the quantum yield of CO_2_ assimilation on the basis of incident light (*Φ*_CO2LL(inc)_, mol CO_2_ mol^-1^ photon) was calculated as:

$\text{}_{CO2LL(inc)}=s^{'}\text{}_{2LL}/4$ (Eq. S6)

where *Φ*_2LL_ (mol e^−^ mol^-1^ photon) and the lumped parameters *s*’ were estimated in the preceding steps.

Finally, with the *Φ*_CO2LL(inc)_ and *R*_d_ estimated above, the maximum leaf photosynthetic rate at the saturating incident irradiance level (*A*_max_, *μ*mol CO_2_ m^-2^ s^-1^) and the curvature factor of the light response curve (*θ*) were estimated by fitting the gas exchange measurements to the non-hyperbola rectangular equation below:

$A=\frac{\text{}_{CO2LL(inc)}I_{inc}+A_{max}-\sqrt{{(\text{}_{CO2LL(inc)}I_{inc}+A_{max})}^{2}-4\text{}A_{max}\text{}_{CO2LL(inc)}I_{inc}}}{2\text{}}-R_{d}$ (Eq. S7)

**References**

**Genty B, Briantais J-M, Baker NR**. **1989**. The relationship between the quantum yield of photosynthetic electron transport and quenching of chlorophyll fluorescence. *Biochimica et Biophysica Acta* **990**: 87–92.

**Yin X, Belay DW, van der Putten PEL, Struik PC**. **2014**. Accounting for the decrease of photosystem photochemical efficiency with increasing irradiance to estimate quantum yield of leaf photosynthesis. *Photosynthesis Research* **122**: 323–335.

**Yin X, Struik PC, Romero P, Harbinson J, Evers JB, Van Der Putten PEL, Vos J**. **2009**. Using combined measurements of gas exchange and chlorophyll fluorescence to estimate parameters of a biochemical C3 photosynthesis model: A critical appraisal and a new integrated approach applied to leaves in a wheat (Triticum aestivum) canopy. *Plant, Cell and Environment* **32**: 448–464.
